# *Candida albicans* Fungaemia following Traumatic Urethral Catheterisation in a Paraplegic Patient with Diabetes Mellitus and Candiduria Treated by Caspofungin

**DOI:** 10.1155/2013/693480

**Published:** 2013-10-08

**Authors:** Subramanian Vaidyanathan, Bakul Soni, Peter Hughes, Gordon Ramage, Leighann Sherry, Gurpreet Singh, Paul Mansour

**Affiliations:** ^1^Regional Spinal Injuries Centre, Southport and Formby District General Hospital, Town Lane, Southport PR8 6PN, UK; ^2^Department of Radiology, Southport and Formby District General Hospital, Town Lane, Southport PR8 6PN, UK; ^3^Infection & Immunity Research Group, School of Medicine, College of Medical, Veterinary and Life Sciences, University of Glasgow, Glasgow G2 3JZ, UK; ^4^Department of Urology, Southport and Formby District General Hospital, Town Lane, Southport PR8 6PN, UK; ^5^Department of Cellular Pathology, Southport and Formby District General Hospital, Town Lane, Southport PR8 6PN, UK

## Abstract

A 58-year-old paraplegic male, with long-term indwelling urethral catheter, developed catheter block. The catheter was changed, but blood-stained urine was drained intermittently. A long segment of the catheter was seen lying outside his penis, which indicated that the balloon of Foley catheter had been inflated in urethra. The misplaced catheter was removed and a new catheter was inserted correctly. Gentamicin 160 mg was given intravenously; meropenem 1 gram every eight hours was prescribed; antifungals were not given. Twenty hours later, this patient developed distension of abdomen, tachycardia, and hypotension; he was not arousable. Computed tomography of abdomen revealed inflamed uroepithelium of right renal pelvis and ureter, 4 mm lower ureteric calculus with gas in right ureter proximally, and vesical calculus containing gas in its matrix. Urine and blood culture yielded *Candida albicans*. Identical sensitivity pattern of both isolates suggested that the source of the bloodstream infection was most likely urine. Both isolates formed consistently high levels of biofilm formation *in vitro* as assessed using a biofilm biomass stain, and high levels of resistance to voriconazole were observed. Both amphotericin B and caspofungin showed good activity against the biofilms. HbA1c was 111 mmol/mol. This patient was prescribed human soluble insulin and caspofungin 70 mg followed by 50 mg daily intravenously. He recovered fully from candidemia.

## 1. Introduction

Candidal colonisation of mucosal sites ordinarily poses no threat to the health of the host. Problems develop when the body's defenses are abridged as occurs with diabetes mellitus, human immunodeficiency virus infection, neutropenia, and immunosuppression accompanying organ transplantation or when patients undergo certain procedures, such as bladder catheterisation or urologic surgery. Breaches in defense allow increased colonisation of mucosal surfaces and sometimes candidemia, in which case the organism can be carried to the kidneys. These predisposing conditions permit the survival of blood-borne or locally invasive yeast in sufficient numbers to evade the local or systemic immunity [1].

Medical devices such as stents and catheters have been shown to support colonisation and biofilm formation by *Candida* spp. [2]. The *Candida* biofilm lifestyle results in antifungal drug resistance and protection of the fungus from host defenses [3]. Candidemia is frequently associated with the biofilm growth of *Candida* organisms on medical devices such as a venous catheter or urinary catheter. In mice infected with *Candida albicans*, degree of biofilm formation was associated with enhanced virulence. Histology of kidney demonstrated massive accumulation of yeast and hyphal elements in renal cortex, medulla, and the papilla associated with degraded glomerulus and renal tubules in high-biofilm forming *Candida* infected mice. In contrast, kidney of low-biofilm forming *C. albicans* infected mice showed infiltration of fungal element and leukocytes mainly in the collecting ducts of the papilla and only few in renal cortex and medullary region with small inflammatory foci [4]. Indwelling urinary catheters and stents are often associated with formation of biofilms. *C. albicans* in biofilms is thought to be recalcitrant to fluconazole, and only lipid formulation of amphotericin and echinocandins have *in vitro* efficacy against *Candida* biofilms [5]. We report *Candida* bloodstream infection following traumatic catheterisation in a spinal cord injury patient, who had uncontrolled diabetes mellitus and *C. albicans* in urine.

## 2. Case Presentation

A 47-year-old British male fell down a height of 40 feet in 2002 and sustained bilateral pneumothoraces, multiple bilateral rib fractures, and multiple, comminuted fractures to the thoracic spine. He developed paraplegia with sensory level of T7. This patient required ventilator support, percutaneous, tracheostomy, and percutaneous endoscopic gastrostomy. Subsequently, this patient was weaned off the ventilator. He had been managing his bladder by long-term indwelling urethral Foley catheter.

In 2006, this patient developed erythema of right foot with purple and necrotic areas at tips of toes and heel. In view of irreversible ischaemia of right foot, right below knee amputation was carried out. In 2009, computed tomography angiogram revealed atherosclerotic changes of aorta and lower limb arteries. There was occlusion of left superficial femoral artery at the level of Hunter's canal, extending down to the trifurcation. In 2010, left above knee amputation was performed for chronic left leg ischaemia. 

In 2012, full blood count showed polycythaemia; haematocrit was 0.61, haemoglobin was 19.5 g/dL, RBC was 6.35 × 10^12^/L, and erythropoietin was 8.5 U/L (reference range: 3.0–18.0). This patient had chronic obstructive pulmonary disease; he was a smoker; he was treated for sleep apnoea with a continuous positive airway pressure machine at home. Diagnosis was secondary polycythaemia; however, blood studies were done to rule out primary bone marrow problem. Genetic analysis for myeloproliferative disorder JAK2 p.V617F mutation in DNA was negative. With this significant polycythaemia, one unit of venesection was performed.

Although this patient had undergone several blood tests, glucose level or HbA1c was not tested after October 2006. In 2013, the urethral catheter was blocked and it was changed by a community health professional at 0200 hours. Following change of catheter, he drained urine only intermittently; the urine was blood stained, and there was blood in the urethral meatus as well. Therefore, this patient came to spinal unit in the morning. On examination, a very long segment of the catheter was lying outside the penis; this indicated that the balloon of Foley catheter had been inflated in the urethra. The misplaced catheter was removed, and a catheter was inserted per urethra correctly. About 350 mL of bloody urine was drained. Gentamicin 160 mg was administered intravenously; meropenem 1 gram every eight hours was prescribed. No antifungals were given to this patient. Blood tests revealed the following: haemoglobin, was 15.1 g/dL, white cell count was 17.3, neutrophils was 16.6, HbA1c was 111 mmol/moL, C-reactive protein was 260.5 mg/L, urea was 6.9 mol/L, creatinine was 85 umol/L.

**Figure 1:**
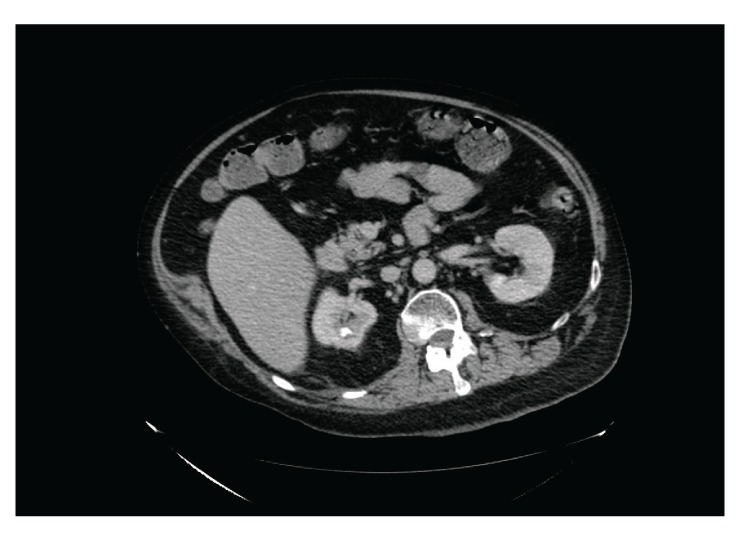
Axial section of computed tomography of abdomen and pelvis revealed 10 mm calculus in upper pole of right kidney.

**Figure 2:**
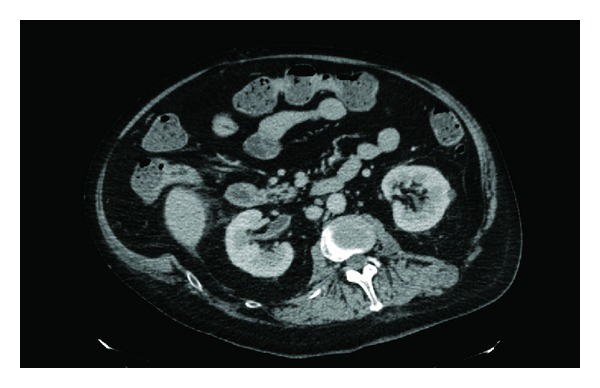
Axial section of computed tomography of abdomen and pelvis revealed inflamed uroepithelium of right renal pelvis and right ureter.

**Figure 3:**
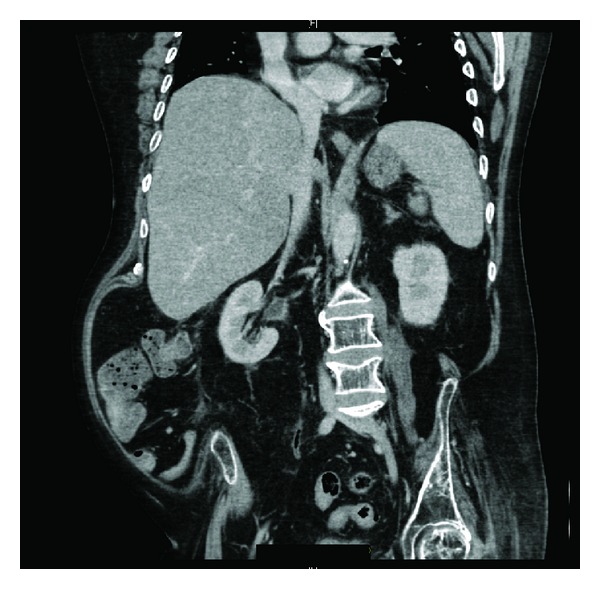
Coronal section of computed tomography of abdomen and pelvis revealed inflamed uroepithelium of right renal pelvis and gas in the lumen of right lower ureter.

**Figure 4:**
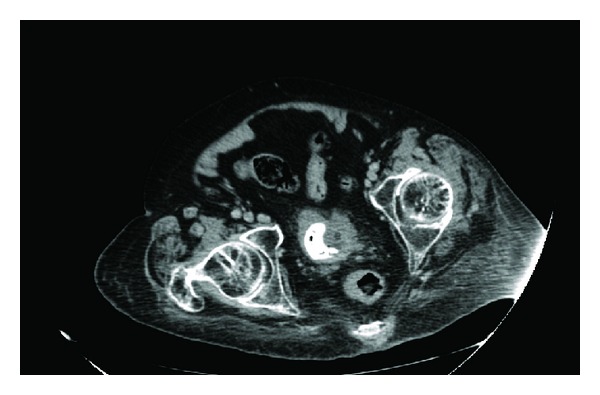
Axial section of computed tomography of abdomen and pelvis: a 31 mm calculus was present in bladder; the stone contained gas in its matrix. Findings of gas within the matrix of vesical calculus indicated that urine infection due to gas forming organisms such as *Escherichia coli* or *Candida albicans* had been present for some time.

**Figure 5:**
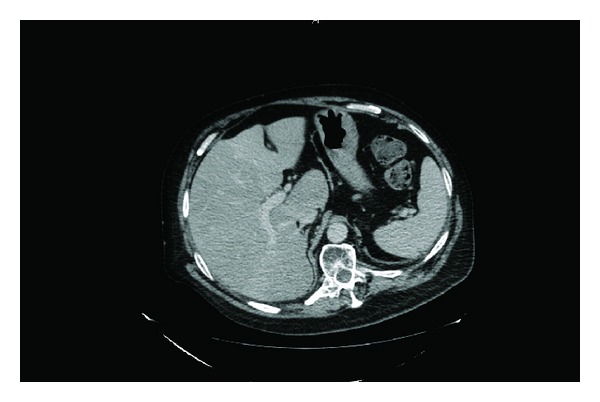
Axial section of computed tomography of abdomen revealed mild hepatomegaly and fatty changes in liver with focal sparing.

Twenty hours after traumatic catheterisation this patient developed distension of abdomen; heart rate increased to 125; blood pressure decreased to 93/52 mm Hg; oxygen saturation dropped to 90%; he could not be aroused to be given medicines or nebuliser. Urine and blood cultures were taken. In view of distended abdomen, computed tomography of abdomen was performed. Computed Tomography revealed the following: 10 mm calculus in upper pole of right kidney (Figure 1), a tiny calculus in the lower pole of the right kidney, inflamed uroepithelium of right renal pelvis and right ureter (Figure 2), 4 mm calculus in lower right ureter with gas in the lumen of right ureter proximally (Figure 3), 31 mm calculus in bladder containing gas in its matrix (Figure 4). There was a minimal right ureteric dilatation. No calculi were seen in the left kidney. A drainage catheter was *in situ* in the bladder. Fatty changes with focal sparing were observed in the mildly enlarged liver (Figure 5). There was no free gas nor fluid in the abdomen or pelvis and no evidence of intestinal obstruction. Umbilical and supraumbilical hernias were present. Radiological evidence of (1) inflammation of right renal pelvis and right ureter, (2) gas in the lumen of right ureter, and (3) gas within the matrix of vesical calculus was consistent with severe infection of urinary tract due to gas forming organism such as *Escherichia coli*, *Klebsiella pneumoniae*, or *C. albicans *[6]. 

Twenty-four hours later, urine microbiology report was received, which revealed growth of >10^8^/L of *C. albicans*. Blood culture report was received forty-eight hours later; blood culture also showed growth of *C. albicans*. In view of positive blood culture, this patient was prescribed human soluble insulin and caspofungin 70 mg on first day followed by 50 mg once daily. 

Fungal isolates from blood and urine were sent to Mycology Reference Laboratory, Wythenshawe Hospital in Manchester, United Kingdom, for sensitivity testing. *C. albicans* isolated from urine was shown to be sensitive to flucytosine, fluconazole, amphotericin, caspofungin, micafungin, and anidulafungin. *C. albicans *grown from blood showed an identical sensitivity pattern; the fungal isolate was sensitive to flucytosine, fluconazole, amphotericin, caspofungin, micafungin, and anidulafungin. The identical sensitivity pattern of both isolates suggested that the source of the bloodstream infection was most likely urine. Isolates from the bloodstream and from the catheter were tested for their ability to form biofilms and their sensitivities were evaluated using fluconazole, amphotericin B, and caspofungin using established methodologies [7, 8]. These data showed that both isolates formed consistently high levels of biofilm formation *in vitro *as assessed using a biofilm biomass stain and high levels of resistance to voriconazole were observed (>64 mg/L). Both amphotericin B (MIC_50_ = 1-2 mg/L) and caspofungin (MIC_50_ = 0.25 mg/L) showed good activity against the biofilms, killing over 50% of the biofilms at these concentrations.

On the fourth day of treatment with caspofungin, blood test revealed a rise in gamma glutamyl transferase level from 314 U/L to 519 U/L. Alkaline phosphatase increased from 114 U/L to 293 U/L. Bilirubin level increased from 6 umol/L to 30 umol/L. Caspofungin was discontinued after six days; this patient was then prescribed fluconazole 400 mg daily by mouth. A week later, alkaline phosphatase level decreased to 174 U/L; gamma glutamyl transferase level also went down to 236 U/L; bilirubin level diminished to 14 umol/L.

48 hours after starting caspofungin, urine showed growth of *C. albicans*; blood culture taken on third day following caspofungin therapy showed no growth; urine showed no growth of *C. albicans* on eighth day after caspofungin therapy. This patient's condition improved and he was discharged home ten days after starting antifungal therapy. 

This patient did not develop any new eye symptoms such as blurring of vision or redness of eyes following this bloodstream infection. He had watering of left eye; but this symptom had been present for quite some time. Ophthalmic examination revealed no evidence of endophthalmitis in both eyes.

Following discharge, this patient was prescribed metformin 500 mg twice a day; insulin was discontinued. Twenty-five days after commencing treatment for diabetes mellitus, HbA1c was 85 mmol/moL; this value of HbA1c indicated poor control of diabetes mellitus. Urine microbiology revealed growth of coliform species and *Enterococcus* species. Urine cytology revealed abundant mixed inflammatory cells, bacteria, benign squamous epithelial cells, scattered, scanty urothelial cells, and macrophages; no malignant cells were seen.

## 3. Discussion

Ang and associates defined potential risk factors for fungaemia as (i) administration of antimicrobial therapy for at least one week before the onset of fungaemia; (ii) administration of corticosteroid therapy at the equivalent of 15 mg of prednisolone daily for at least one week; (iii) administration of cytotoxic therapy; (iv) manipulation of urinary tract within 2 weeks of the onset of fungaemia [9].

Urologic procedures, which can predispose to fungaemia, include open surgery, nephrostomy, cystoscopy, stent placement, intermittent urinary catheterisation, and placement of an indwelling urinary catheter. In contrast to candidemia that arises from an intravascular focus, episodes of candiduria-associated candidemia were of low grade and of short duration and in many cases resolved spontaneously prior to the institution of specific antifungal therapy. Patients with candidemia from a urinary source had a characteristic profile: most of them had significant anatomical urinary tract pathology that was complicated by obstruction; these patients were subjected to urological procedures. Although a few patients became candidaemic within 24 hours after undergoing a urological procedure, candidemia in most patients did not occur immediately after manipulation of urinary tract but several days later. A plausible explanation for this course of events includes the following factors: (1) an ascending infection facilitated by urinary manipulation in the presence of candiduria; (2) subsequent development of micro- or macrofoci of renal parenchyma infection; and (3) limited or transient low-grade candidemia, perhaps precipitated by additional insults. Our patient developed features of sepsis 24 hours after traumatic catheterisation. Urine and blood cultures were then taken; both urine and blood cultures showed growth of *Candida albicans*. We believe that traumatic catheterisation in the presence of candiduria facilitated (i) an ascending urinary tract infection, as shown by inflamed uroepithelium of right renal pelvis and ureter, and (ii) dissemination of *Candida* into bloodstream through damaged bladder and urethral mucosa. 

Liver cirrhosis has been occasionally cited as a risk factor for fungaemia after urological procedures. Toshikuni and associates reported a case of fungal sepsis and *C. albicans* endophthalmitis after extracorporeal shock wave lithotripsy in a cirrhotic patient [10]. Beck and associates reported two patients with Child-Pugh class B and C liver cirrhosis who had undergone ureteroscopy and holmium laser lithotripsy for obstructing ureteral calculi [11]. Within 12 hours of ureteroscopy, both patients became tachycardic, hypotensive, and febrile. Blood, urine (proximal to the stone), and stone cultures were positive for *C. albicans* in both patients. Both patients were treated successfully with intravenous fluconazole. Cirrhotic patients have decreased opsonin function, an impaired complement system, decreased antibody production, and decreased tumour necrosis factor levels. Hassner and associates demonstrated that monocytes of cirrhotic patients have lower phagocytic uptake and killing of *Candida* strains compared with that of controls [12]. Our patient reported here had elevated level of gamma glutamyl transferase (314 U/L; reference range: 0–50) prior to developing fungaemia, which might be indicative of some degree of liver dysfunction. In this patient with uncontrolled diabetes mellitus, computed tomography revealed mild hepatomegaly and fatty changes in liver with focal sparing. A further increase in gamma glutamyl transferase and a rise of alkaline phosphatase level (from 114 U/L to 293 U/L) were observed following intravenous caspofungin therapy. caspofungin was discontinued and fluconazole by mouth was prescribed in order to facilitate discharge from the hospital. Fluconazole was less expensive than caspofungin and high cost of caspofungin could not be ignored. However, it should be remembered that Fluconazole therapy can result in liver toxicity, which ranges from mild and transient enzyme elevations to clinically apparent hepatitis to acute liver failure and death. The cause of clinically apparent hepatotoxicity from fluconazole is unknown; however, it may relate to the ability of fluconazole to alter sterol synthesis. Fluconazole is a potent inhibitor of the cytochrome P450 enzyme CYP3A4 and can lead to significant increases in plasma levels and serious toxicity from medications that are ordinarily metabolized by CYP3A4, particularly the statins and cyclosporine [13]. 

Microbial biofilms develop when organisms adhere to a surface and produce extracellular polymers that provide a structural matrix and facilitate further adhesion [3, 5, 14]. Organisms in biofilms behave differently from freely suspended microbes and have been shown to be relatively refractory to medical therapy. *C. albicans* biofilm formation has three developmental phases: adherence of yeast cells to the device surface (early phase), formation of a matrix with dimorphic switching from yeast to hyphal forms (intermediate phase), and increase in the matrix material taking on a three-dimensional architecture (maturation phase) [15]. Fully mature *Candida* biofilms have a mixture of morphological forms and consist of a dense network of yeasts, hyphae, and pseudohyphae in a matrix of polysaccharides, carbohydrate, protein, and unknown components [14]. The formation and structure of these biofilms are influenced by the nature of the contact surface, environmental factors, morphogenesis, and the *Candida* species involved. 

Tumbarello and associates identified two unique risk factors, diabetes mellitus and urinary catheterisation, which were specifically associated with biofilm-forming *Candida* bloodstream infection [5]. Candidemia is frequently associated with the biofilm growth of *Candida* organisms on medical devices such as a venous catheter or urinary catheter [2]. Such infection is serious because biofilms are thought to be recalcitrant to antifungals, for example, fluconazole, while only two classes of agents (i.e., liposomal amphotericin B and echinocandins) appear to have *in vitro* efficacy against *Candida *[16].

Tumbarello and colleagues reported that clinical isolates from *Candida* bloodstream infections that formed biofilms displayed greater levels of morbidity and mortality [17]. Therefore, it may be prudent to test and establish whether or not clinical isolates of *Candida *from selected patients form biofilms: for example, those who develop *Candida* bloodstream infection associated with a central venous line or a peripherally inserted central catheter, implanted medical devices such as joint prostheses, or indwelling urinary catheter. If biofilm formation is detected, such a patient will require appropriate antifungal therapy. Kojic and Darouiche reported that *C. albicans* in biofilms on polyvinyl chloride disks is 30 to 2,000 times more resistant to fluconazole, amphotericin B, flucytosine, itraconazole, and ketoconazole than planktonic cells [18], and the biofilm structure remained intact at an amphotericin B concentration of 11 times the minimum inhibitory concentration. However, both echinocandins and liposomal formulations of amphotericin B have been shown to be effective against *C. albicans* biofilms [16, 19, 20]. Suggested mechanisms of biofilm resistance include restricted penetration of drugs through the matrix, slow growth of organisms in biofilms accompanied by changes in the cell surface composition affecting their susceptibility to drugs, and unique biofilm-associated patterns of gene expression [14]. 

Toshikuni and associates suggested that administration of antifungal agents before extracorporeal shock wave lithotripsy might be helpful for preventing fungaemia in patients with funguria [10]. The mechanical trauma to the ureter during extracorporeal shock wave lithotripsy might result in dissemination of *C. albicans* into the bloodstream from urine, infected stent, or urinary calculi. It appears reasonable to extend this concept of administering antifungals to spinal cord injury patients with candiduria, in whom urethral catheterisation proves traumatic, as indeed happened to our patient. 

## 4. Learning Points from This Case

- Antifungal therapy with echinocandins or lipid formulation of amphotericin B should be administered after traumatic catheterisation in a spinal cord injury patient with uncontrolled diabetes mellitus and *C. albicans* in urine.
- Fungal isolates may be tested for biofilm formation capacity, as infection with biofilm-forming *Candida* species is recalcitrant to fluconazole; only lipid formulation of amphotericin B and echinocandins appear to have *in vitro* efficacy against *Candida* biofilms.
- Persons with spinal cord injury are at increased risk for developing diabetes mellitus. This patient was not tested for diabetes mellitus during the past twelve months although he visited the hospital several times. This case is a reminder that spinal injury patients require global assessment instead of fragmented care focusing on just one aspect such as management of respiratory tract or pressure sores.
- In this patient, who had raised gamma glutamyl transferase and alkaline phosphatase, fluconazole was prescribed to facilitate discharge home. Spinal cord physicians should remember that fluconazole therapy can result in liver toxicity, which ranges from mild and transient enzyme elevations to clinically apparent hepatitis to acute liver failure and death.
- Computed tomography of urinary tract proved to be a valuable investigation in this patient. CT revealed evidence of inflammation of right renal pelvis and ureter and gas in right ureter and in the matrix of vesical calculus.
